# Developmental Patterns of Doublecortin Expression and White Matter Neuron Density in the Postnatal Primate Prefrontal Cortex and Schizophrenia

**DOI:** 10.1371/journal.pone.0025194

**Published:** 2011-09-26

**Authors:** Samantha J. Fung, Dipesh Joshi, Katherine M. Allen, Sinthuja Sivagnanasundaram, Debora A. Rothmond, Richard Saunders, Pamela L. Noble, Maree J. Webster, Cynthia Shannon Weickert

**Affiliations:** 1 Schizophrenia Research Institute, Sydney, Australia; 2 Neuroscience Research Australia, Sydney, Australia; 3 School of Medical Sciences, University of New South Wales, Sydney, Australia; 4 School of Psychiatry, University of New South Wales, Sydney, Australia; 5 Laboratory of Neuropsychology, National Institute of Mental Health (NIMH), National Institutes of Health, Bethesda, Maryland, United States of America; 6 National Institute of Mental Health (NIMH) IRP Non-Human Primate Core, Poolesville, Maryland, United States of America; 7 Stanley Medical Research Institute, Rockville, Maryland, United States of America; Institut National de la Santé et de la Recherche Médicale, France

## Abstract

Postnatal neurogenesis occurs in the subventricular zone and dentate gyrus, and evidence suggests that new neurons may be present in additional regions of the mature primate brain, including the prefrontal cortex (PFC). Addition of new neurons to the PFC implies local generation of neurons or migration from areas such as the subventricular zone. We examined the putative contribution of new, migrating neurons to postnatal cortical development by determining the density of neurons in white matter subjacent to the cortex and measuring expression of doublecortin (DCX), a microtubule-associated protein involved in neuronal migration, in humans and rhesus macaques. We found a striking decline in DCX expression (human and macaque) and density of white matter neurons (humans) during infancy, consistent with the arrival of new neurons in the early postnatal cortex. Considering the expansion of the brain during this time, the decline in white matter neuron density does not necessarily indicate reduced total numbers of white matter neurons in early postnatal life. Furthermore, numerous cells in the white matter and deep grey matter were positive for the migration-associated glycoprotein polysialiated-neuronal cell adhesion molecule and GAD65/67, suggesting that immature migrating neurons in the adult may be GABAergic. We also examined DCX mRNA in the PFC of adult schizophrenia patients (n = 37) and matched controls (n = 37) and did not find any difference in DCX mRNA expression. However, we report a negative correlation between DCX mRNA expression and white matter neuron density in adult schizophrenia patients, in contrast to a positive correlation in human development where DCX mRNA and white matter neuron density are higher earlier in life. Accumulation of neurons in the white matter in schizophrenia would be congruent with a negative correlation between DCX mRNA and white matter neuron density and support the hypothesis of a migration deficit in schizophrenia.

## Introduction

Interstitial white matter neurons (IWMNs) are a population of neurons that reside among the fibres and glia of white matter, particularly in primates [1], [2], [3]. These neurons are present in adult animals and are thought to be remnants of the subplate, a transient layer below the cortical plate in the developing brain that provides guidance and a temporary target for thalamocortical axons. Subplate neurons are also important in the development of cortical columns and maturation of inhibitory circuitry [4], [5], [6], [7] and many of these subplate cells undergo apoptosis during normal development. Some IWMNs persist in the adult [8], [9] however their function in the mature brain is not understood, although a role in vasodilation and vasoconstriction has been suggested [10], [11]. It has also been suggested that some IWMNs may be neurons of subventricular zone (SVZ) origin originally destined for the cortex, however they remain in the white matter [12]. Interestingly, in schizophrenia there is an increased density of IWMNs [13], [14], [15], [16], [17], [18] which might indicate a deficit in the cell death of subplate neurons and/or deficient migration of neurons during development or at maturation.

Doublecortin (DCX) is a microtubule associated protein expressed in immature, migrating neurons [19], [20]. DCX has been identified as an important molecule in the proper lamination of the cortex, with mutations in the *Dcx* gene causing lissencephaly or double cortex syndrome in humans [21], [22]. Such dramatic phenotypes are not present in mouse models [23], [24] likely due to redundancy with doublecortin-like (DCL) and DCX-like kinase (DCLK) [25], [26]; however, RNAi knockdown of DCX results in migrational deficits in the rostral migratory stream or in tangentially migrating interneurons destined for the cortex [26], [27], [28], [29], [30]. DCX expression in new neurons is thought to be transient [31] with expression beginning approximately 1 day following the birth of a new cell and being maintained for 2–3 weeks after this time when cells begin to down-regulate DCX and up-regulate markers of mature neurons (eg NeuN) [32], such that DCX and NeuN may be co-expressed in the developing neuron [33], [34]. DCX therefore represents an endogenous marker of immature, migrating neurons. In addition to DCX, the presence of polysialic acid on neural cell adhesion molecule (PSA-NCAM) reduces cell-cell interactions and plays an important role in cellular plasticity as well as being required for the migration of neuroblasts in the rostral migratory stream (reviewed by [35]), making PSA-NCAM another marker of immature, migrating neurons.

Our previous studies have estimated that thousands of immature neurons that express markers of migration, arranged in clusters adjacent to the SVZ are present in the human infant [36], [37] and while the olfactory bulb may be among their targets, their final destination is unknown. As cortical grey matter volume increases from birth to about 5 years of age [38], [39], [40], [41], we hypothesise that the recruitment of new, migrating neurons may contribute to postnatal cortical growth. In this study, we addressed whether new neurons could contribute to increasing postnatal cortical volume by examining whether IWMNs may represent a population of migrating neurons in the postnatal primate brain, and determining whether neuronal migration could be detected in the overlying dorsolateral prefrontal cortex (DLPFC) during postnatal development by analysing DCX expression [19], [32]. We demonstrate that NeuN+ IWMN density and DCX expression are at elevated levels in the early human and rhesus macaque DLPFC prior to reaching adult levels, implying a robust recruitment of new neurons to the primate cortex in early life.

In addition, several lines of evidence suggest that altered neurogenesis may be linked to schizophrenia. Indeed, many schizophrenia-associated genes such as neuregulin-1, ErbB4, and reelin play a role in neuronal differentiation and migration (see recent reviews by [42], [43]). A reduction in cells positive for markers of cell division or neuronal migration, Ki67 and PSA-NCAM, has also been reported in the hippocampus of people with schizophrenia [44], [45]. Therefore, a second aim of this study was to test if altered DCX mRNA, as a marker of neuronal migration, was changed in the frontal cortex of people with schizophrenia and relate DCX expression in the grey matter to IWMN density in the white matter, that we have previously found to be increased in this schizophrenia cohort [18].

## Materials and Methods

### Ethics statement

All non-human primate research procedures were carried out in strict adherence to the laws and regulations of the U.S. Animal Welfare Act, (USDA, 1990) and Public Health Service Policies, (PHS, 2002) as well as non-governmental recommendations of the National Research Council as published in the ILAR “Guide for the Care and Use of Laboratory Animals”. All research facilities were approved by the International Association for the Assessment and Accreditation of Laboratory Animal Care. The work was carried out under an Animal Study Protocol approved by the NIMH Animal Care and Use Committee. Therefore all research practices were consistent with the recommendations of the Weatherall Report (2006) on “The Use of Non-Human Primates in Research”.

### Human post-mortem brain samples

Developing human post-mortem DLPFC tissue was obtained from the University of Maryland Brain Tissue Bank for Developmental Disorders (NICHHD contract # NO1-HD8-3283). The human developmental cohort consisted of 68 individuals ranging in age from 6 weeks to 49 years (Summarised in Table 1, details of subjects used for all analyses in Table S1). These samples were a priori divided into seven developmental periods: neonates, infants, toddlers, school age, teenagers, young adults and adults, as described previously [46]. Neonates and infants were full-term and all subjects were free of neurological and gross behaviour changes at the time of death [36], [47], [48]. Moreover, toxicological analyses showed them to be free of drug use [36], [47], [48]. Tissue from the DLPFC of patients with schizophrenia/schizoaffective disorder (n = 37) and matched controls (n = 37) was obtained from the New South Wales Tissue Resource Centre (Table 2; Sydney, Australia, HREC 07261). Groups within both cohorts were matched according to tissue pH, PMI, RIN and, in the schizophrenia cohort, age [49].

**Table 1:** Summary of developmental cohort demographics.

Group	Age (years)	Gender	PMI (hours)	pH	RIN	#
**Human**						
Neonate	0.11–0.24	7M 4F	22.45±5.11	6.6±0.19	6.37±1.64	11
Infant	0.25–0.91	8M 6F	16.93±6.4	6.58±0.20	6.93±1.18	14
Toddler	1.58–4.86	5M 4F	18.67±5.29	6.70±0.26	6.51±1.21	9
School age	5.39–12.98	5M 4F	15.11±4.68	6.63±0.27	6.66±1.14	9
Teenage	15–17.82	6M 2F	17.13±4.16	6.75±0.09	6.34±1.01	8
Young adult	20.14–25.38	6M 3F	13.67±8.26	6.67±0.23	6.73±0.67	9
Adult	35.99–49.22	5M 3F	13.38±4.60	6.60±0.27	6.53±0.76	8
**Rhesus macaque**						
Neonate	0.04–0.16	1M 3F			7.41±0.27	4
Infant	0.75–1.33	2M 2F			6.24±0.54	4
Juvenile	2–2.5	2M 3F			6.44±1.03	5
Adolescent	3.08–4.5	10M 3F			6.58±1.28	13
Young Adult	6.33–7.58	6M 2F			7.23±0.18	8
Adult	8–12.08	7M 4F			7.02±0.63	11

**Table 2:** Summary of demographics for control and schizophrenia groups.

	control group (n = 37)	schizophrenia group (n = 37)
Age (years)	51.1 (18–78)	51.3 (27–75)
gender	7F, 30M	13F, 24M
hemisphere	23R, 14L	17R, 20L
pH	6.66±0.29	6.61±0.30
PMI (hrs)	24.8±10.97	28.8±14.07
RIN	7.3±0.57	7.3±0.58
subclass	-	paranoid = 16; undifferentiated = 7; disorganised = 5; residual = 2; schizoaffective, depressive type = 4; schizoaffective, bipolar type = 3
age of onset (years)	-	23.7±0.10
duration of illness (years)	-	27.6±2.3

### Non-human primate brain samples

The non-human primate developmental cohort consisted of 2 week to 12 year old *Macaca mulatta* (rhesus macaques) (n = 45) from the NIMH, NIH (Table 1). Animals were euthanised, flushed with saline and 1 cm coronal sections of brain were flash frozen and stored at −80°C. For fluorescence immunohistochemistry for NeuN and GAD65/67, 14 µm fresh frozen coronal sections from the frontal cortex (containing the principal sulcus) of three adolescent male animals (all 4.5 years old) from the rhesus macaque developmental cohort were used. Fresh frozen coronal sections containing the principal sulcus obtained from three adult male animals (6.5, 7.6 and 9.6 years old) was used for additional PSA-NCAM immunohistochemistry.

Rhesus macaque tissue was also obtained from one 12 day old, 1 month old, 3.7 year old, and 6 year old rhesus macaque for DCX immunohistochemistry. Animals were euthanised, and flushed with saline then 4% paraformaldehyde. Brains were then post-fixed, cryoprotected, frozen and sectioned at a thickness of 40 µm and stored in cryoprotectant solution [25% glycerol, 25% ethylene glycol in phosphate buffered saline (PBS)] at −20°C.

### Immunohistochemistry

#### DAB immunohistochemistry for NeuN in human DLPFC

14 µm sections of the human middle frontal gyrus were cut from frozen tissue blocks using a cryostat (Leica CM3050 S) and thaw-mounted onto gelatin-coated slides. Sections were stored at −80°C and thawed at room temperature (RT) for 20 min prior to immunohistochemistry. Immunohistochemistry for NeuN was performed on 14 µm fresh frozen sections, as previously detailed [18]. Briefly, sections were fixed in 4% paraformaldehyde in PBS (137 mM NaCl, 2.7 mM KCl, 8 mM Na_2_HPO_4_, 2 mM KH_2_PO_4_, pH 7.4), 10 min at 4°C, washed in PBS then endogenous peroxidases were quenched for 20 min at RT with methanol+3% H_2_O_2_ (3∶1) solution. Sections were washed and blocked with 10% normal goat serum in diluent [0.05% bovine serum albumin (BSA), 0.3% triton X-100 in PBS] for 1 hr at RT. Mouse anti-NeuN antibody (Millipore MAB377, 1∶1000 in diluent) was applied overnight at 4°C. Following washing, goat anti-mouse IgG biotinylated secondary antibody (Vector Laboratories, Cat # BA-9200; 1∶500 in diluent) was applied for 1 hr at RT. Slides were washed, incubated at RT (1 hr) in avidin–biotin–peroxidase complex (Vectastain ABC kit; Vector Laboratories) and treated with 3,3′–diaminobenzidine (DAB; Sigma; 12 mM final concentration in PBS with 0.003% H_2_O_2_) for 5–7 min. Slides were Nissl stained (1.5 min exposure to 0.02% thionin) and coverslipped. PSA-NCAM immunohistochemistry was also performed in 14 µm sections of fresh frozen tissue from adult rhesus macaques (6.5, 7.6 and 9.6 years of age) using the DAB method (Table S2). Primary antibodies were omitted as a negative control and did not show immunoreactivity.

#### DAB immunohistochemistry for DCX, GAD65/67 and PSA-NCAM in rhesus macaque

DAB immunohistochemistry for DCX, GAD65/67 and PSA-NCAM was performed on 40 µm rhesus macaque fixed-floating coronal sections at the level of the head of the caudate with antibodies detailed in Table S2. Floating sections were washed in PBS and endogenous peroxidases were quenched with methanol+3% H_2_O_2_ (3∶1) solution, 20 mins at RT, washed, then blocked with 10% goat or rabbit serum for 1 hr at RT. Primary antibodies were applied in diluent at concentrations specified in Table S2 for two nights at 4°C. Sections were washed in PBS and secondary antibodies were applied in diluent, 1 hr at RT, and avidin-biotin complex, DAB reaction and thionin counterstaining were performed as for NeuN immunostaining. Controls were performed where primary antibodies were omitted and were negative for immunoreactivity.

#### Double-label immunohistochemistry for NeuN and GAD65/67 in rhesus macaque

Fluorescence immunohistochemistry to demonstrate co-localisation of NeuN and GAD65/67 was performed on 14 µm thick fresh-frozen sections from rhesus macaque. Tissue was thawed, fixed in 4% paraformaldehyde for 10 min at 4°C, rinsed and blocked with 10% donkey serum in diluent for 1 hr at RT then primary antibodies (1∶1000 mouse anti-NeuN and 1∶500 rabbit anti-GAD65/67) were applied in diluent overnight at 4°C. Following washing, secondary antibodies were applied, each at 1∶1000 dilution (Alexa Fluor 488 donkey anti-mouse IgG, Invitrogen A21202 and Alexa Fluor 594 conjugated donkey anti rabbit IgG, Invitrogen A21207) for 1 hr at RT prior to washing in PBS, then 4′,6-diamidino-2-phenylindole (DAPI, 1∶1000) in PBS for 5 mins and a further wash in PBS before slides were coverslipped. Controls for binding of the secondary antibody were performed where one primary antibody was omitted (ie only anti-NeuN or only GAD65/67 were applied). Alexa Fluor 488 signal was absent, and some faint Alexa Fluor 594 signal was detected, but was below intensity of the signal in the presence of the GAD65/67 antibody.

### Quantification of NeuN+ IWMNs

Images of NeuN immunostaining were captured at 10× magnification and stitched together using the Virtual Slice facility in the Stereo Investigator Software (MBF Biosciences). The grey matter/white matter boundary was identified by a sharp change in NeuN density, the presence of additional smaller astrocytic nuclei and lighter NeuN neuropil staining (Figure S1) as detailed in [18]. IWMN density was quantified around the middle frontal gyrus of cases from the human developmental cohort and data from control cases from the schizophrenia cohort aged between 18 and 50 years [18] were included in this analysis to increase statistical power within the older age groups (Table S1). Regions of white matter sampled were selected on straight banks of white matter avoiding the crown and the curve at the deep end of the sulcus, where IWMN density can be inconsistent. Superficial IWMNs were defined as those lying between 0 and 700 µm deep to the grey/white matter border and 20 sampling boxes 470×470 µm were placed within this region parallel to the grey/white matter border at random distances between each other and from the grey/white matter border within the 700 µm superficial compartment. All NeuN+ IWMNs within boxes were counted, except those touching the right and bottom sides of the counting box. Slides were sampled and counted by two researchers. The final NeuN+ density was calculated as the average of the 20 boxes. Researchers were blind to developmental age group through the experimental and quantification procedure.

### Microarray analysis

Total RNA from human DLPFC tissue (grey matter) was extracted from all subjects using the Trizol method (Invitrogen). RNA quality was assessed using the 2100 Bioanalyser electrophoresis system (Agilent Technologies). RNA was purified through a Qiagen RNA mini Kit (Qiagen Inc, Valencia CA USA) from 45 individuals of the human developmental cohort (Table S1) was prepared for microarray analysis according to Affymetrix protocol (www.affymetrix.com) using HG-U133 version 2.0+ (GeneChips, Affymetrix CA, USA) as described previously [50]. The Bioconductor package was used to compute normalised expression values from the Affymetrix.cel files and statistical analysis was performed using R and Bioconductor software. Probe sets that met the criteria of being 50% present in at least one of the age/gender subgroups were retained in the analysis (33,210 probes sets retained, 61% of total number). All data are MIAME compliant and raw data has been deposited in the GEO database (NCBI) with the accession number GSE13564.

### Quantitative real time PCR analysis

cDNA was synthesised from total RNA extracted from the cortical grey matter (human DLPFC) or frontal pole (rhesus macaque) using the SuperScript® First-Strand Synthesis kit and random hexamers (Invitrogen) from 3 µg of total RNA per sample, repeated twice and pooled. DCX transcript levels were measured by quantitative real time-PCR (qPCR) using an ABI Prism 7900HT Fast Real time PCR system with a 384-well format and TaqMan Gene Expression Assays (Applied Biosystems) (Hs01035496_m1 for human and Rh02829106_m1 for rhesus macaque). All measurements from each subject were performed in triplicate and relative quantities determined from a seven point standard curve. Transcript quantities were normalised by the geometric mean of four housekeeping genes: GUSB (Hs99999908_m1), PBGD (Hs00609297_m1), PPIA (Hs99999904_m1) and UBC (Hs00824723_m1) for the human developmental series and UBC, ACTB (Hs99999903_m1), GAPDH (Hs99999905_m1), TBP (Hs00427620_m1) for the schizophrenia cohort [49] and TBP, SDHA (Hs01549169_m1) and ACTB for the rhesus developmental series.

### Western Blot analysis

Western blot analysis for DCX was performed as previously described [46]. Briefly, 40 mg of pulverised frozen tissue was homogenised in 400 µl of homogenisation buffer [50 mM Tris pH 7.5, 50% glycerol and 1∶20 v/v of protease inhibitor cocktail (Sigma, P8340) final concentration: 2 mM aminoethylbenzenesulfonyl fluoride, 0.015 mM aprotinin, 0.038 mM leupeptin, 0.030 mM, pepstatin A, 0.028 mM E-64, 0.08 mM bestatin]. Protein concentration per sample was determined using Bradford (Sigma) and BCA (Thermo Scientific) protein assays. Analyses were performed in duplicate with 12 µg of total protein for each human sample and 30 µg total protein for each rhesus macaque sample, heat denatured at 95°C in 1 volume of Lamelli buffer (BioRad) with 0.5% β-mercaptoethanol, analysed by SDS-PAGE on a 4–12% Bis-Tris gel (BioRad) and transferred onto PVDF membrane (BioRad) for 1 hr on ice. 10 µl of Dual colour Precision Plus Protein Prestained Standard (BioRad) was loaded on each gel. Membranes were blocked (5% w/v non-fat milk, 0.1% v/v Tween-20 in PBS) for 1 hr at RT with agitation, then incubated with goat polyclonal anti-DCX (1∶200, sc-8066, Santa Cruz Biotechnology) primary antibody in 1% w/v non-fat milk, 0.1% v/v Tween-20 in PBS overnight at 4°C with agitation. Donkey anti-goat horse-radish peroxidase (HRP)-conjugated secondary antibody (1∶10 000, sc-2033, Santa Cruz Biotechnology) secondary antibody in 1% w/v non-fat milk, 0.1% v/v Tween-20 in PBS, was applied for 1 hr at RT. Bands were visualised using chemiluminescent HRP substrate (Immobilon™ Western, Millipore) and quantified by densitometry using Image J [51]. As a loading control, membranes were probed with mouse anti-actin (1∶10,000; Chemicon; AB1501), followed by goat anti-mouse HRP-conjugated secondary antibody (1∶5,000). Average intensity for each sample was normalised with the respective β-actin average intensity and an internal control (protein from human neonate and adult tissue or a pooled sample of all rhesus macaque developmental cohort cases) from each gel.

### Statistical analysis

No group outliers of IWMN density were detected in any of the human developmental groups by Grubbs test. Microarray data examining gene expression across age were analysed in a linear regression model including age. Samples with RINs less than 5.8 were excluded (n = 11) from developmental qPCR data and outliers within a developmental group (significant, p<0.05 by Grubb's method) or two standard deviations from the mean for the schizophrenia cohort were removed for ANOVA/ANCOVA (<5%). ANOVA was performed with Fisher-LSD post hoc analyses to determine differences in IWMN density or DCX mRNA/protein between developmental groups. Pearson's correlations were performed to examine the relationship between DCX mRNA expression and IWMN density in the human. Statistical tests were performed using Statistica software (version 7.1) and data are reported as mean ± standard deviation.

## Results

### IWMN density under the middle frontal gyrus declines over postnatal age in the human

**Figure 1:**
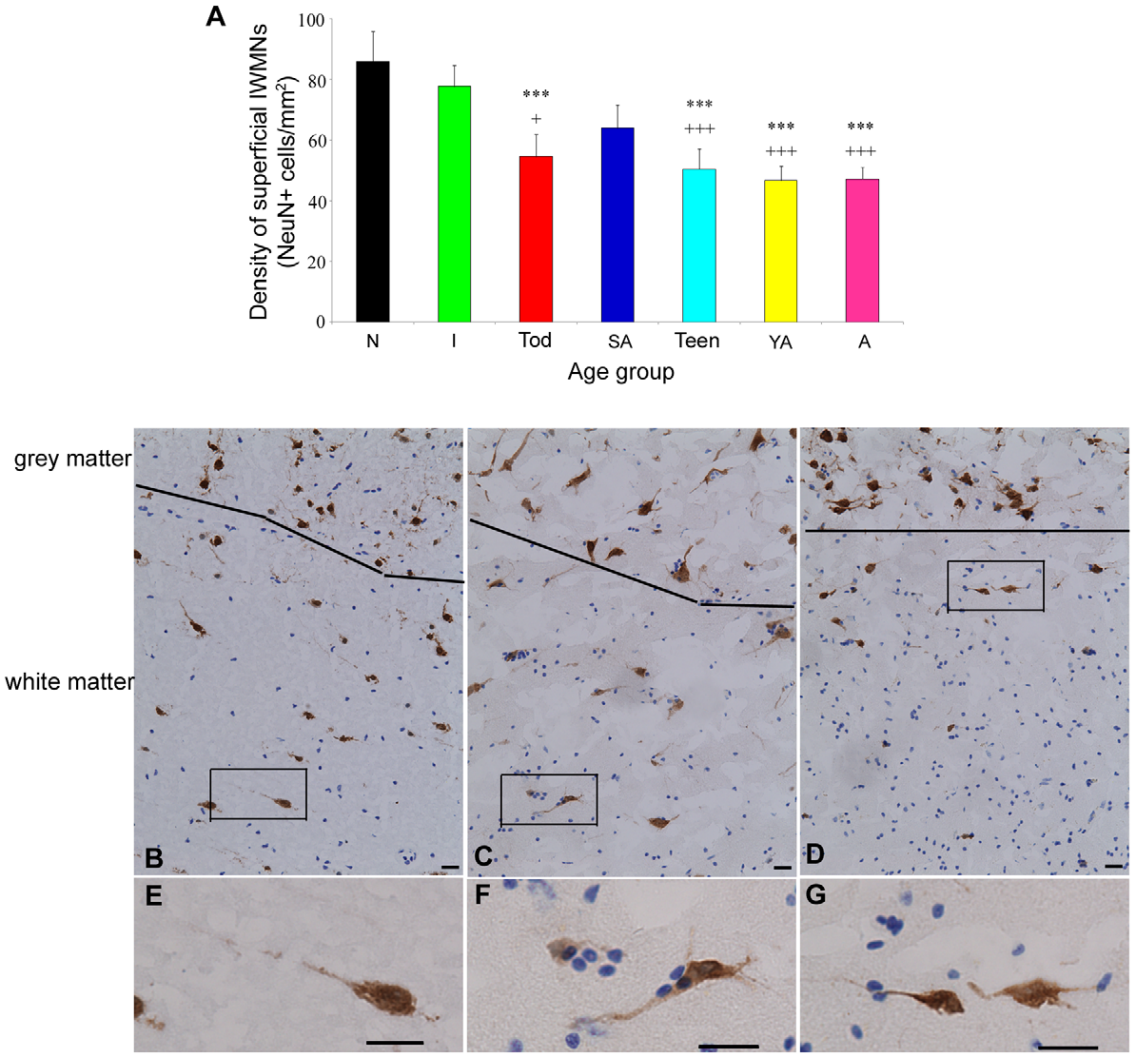
Interstitial white matter neuron (IWMN) density declines over postnatal development. (**A**) The mean density of NeuN immunopositive IWMNs in the superficial white matter of the middle frontal gyrus was quantified in different developmental age groups: neonate (N, n = 7), infant (I, n = 12), toddler (Tod, n = 8), school age (SA, n = 6), teenage (Teen, n = 8), young adult (YA, n = 8), adult (A, n = 15). ***p<0.005 compared to neonates; +p<0.05, +++p<0.005 compared to infants. Error bars represent standard error. (**B**–**D**) Representative photos of NeuN+ IWMNs in (**B**, inset **E**) neonate, (**C**, inset **F**) school aged, and (**D**, inset **G**) adult individuals, line represents approximate grey matter/white matter border. Scale bars = 20 µm.

The density of NeuN+ IWMNs showed a significant developmental change (ANOVA F = 5.6, df = 57, p = 0.0001, Figure 1A) being highest in the neonate group (85.9±26.4 NeuN+ cells/mm^2^, representative image shown in Figure 1B) and being significantly reduced by 36–46% in toddlers, teenagers, young adults and adults (all p<0.005 compared to neonates and infants, with the exception of toddlers and infants, being p = 0.01 by Fisher LSD post hoc analysis). Interestingly the NeuN+ cell density in school age individuals (Figure 1C) is not significantly different from infants, but shows a trend to be reduced in school aged individuals compared to neonates (25.5% fewer cells, p = 0.053) and a trend to be elevated relative to adults (26.3% more cells, p = 0.09, representative image in Figure 1D) which may indicate that IWMN density might increase slightly in the school age period (6–13 years of age) prior to puberty.

### Expression of DCX dramatically declines over early human postnatal development

**Figure 2:**
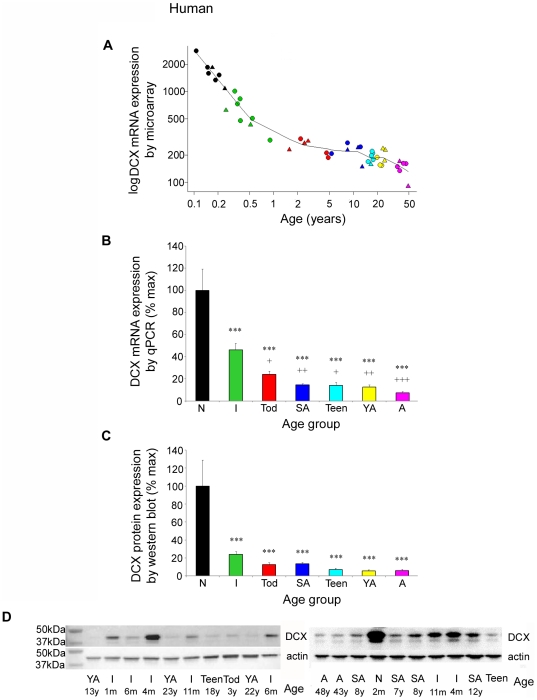
Doublecortin (DCX) is downregulated in the DLPFC of human brain during postnatal development. (**A**) mRNA expression profile of DCX across chronological age by microarray (males = circles, females = triangles). (**B**) The developmental profile of DCX mRNA expression was replicated by qPCR [DCX mRNA expression (mean ± SEM) was normalised to the geometric mean of four housekeeper genes] (**B**), and (**C**) at the protein level [expression (mean ± SEM) normalised to β-actin]. (**D**) Representative western blot for DCX and β-actin in individuals from different developmental age groups. ***p<0.001 compared to neonate; +p<0.05, ++p<0.005, +++p<0.001 compared to infants. Black, neonates (N); green, infants (I); red, toddlers (Tod); dark blue, school age children (SA); light blue, teenagers (Teen); yellow, young adults (YA); pink, adults (A).

By microarray analysis, DCX (probe set 204850_s_at) showed a dramatic reduction in mRNA levels in the human DLPFC early in postnatal life with the largest decrease (p<0.001, r = 0.925, Figure 2A) in transcript levels from newborns to mature adults. The relatively high expression in neonates compared to adults represents the largest and most significant change in gene expression found in the developing human brain with age out of the 55,000 transcripts surveyed on the Affymetrix U133A chip [50]. The extent and timing of this marked reduction in developmental DCX expression was confirmed by qPCR analysis, with an approximately 94% reduction between neonates and adults for DCX mRNA (ANOVA F(6, 50) = 15.5, p<0.00001, Figure 2B). A significant difference was noted in DCX expression levels between neonates/infants and the rest of the developmental age groups (p<0.00001 for neonates, p<0.05 for infants) demonstrating that the most dramatic change in expression takes place within the first postnatal year of human life. Interestingly, DCX mRNA levels are maintained throughout adult life at a level above the limit of detection. Using Western blot analysis, we observed a marked reduction in DCX protein levels across the developmental period examined with an approximate 94% reduction between neonates and adults (ANOVA F(6, 51) = 11.4, p<0.000001, Figure 2C), with the most prominent band at the predicted size of 40 kDa which was strongly expressed in younger individuals (up to 13 years of age) and weakly expressed in older age groups such as older teens, young adults and adults (Figure 2D). Variability in band intensity was particularly evident in the infant group (Figure 2D), which may be attributed to the large change in expression over the first year of life, demonstrated in Figure 2A.

### Expression of DCX in the developing rhesus macaque primate brain

**Figure 3:**
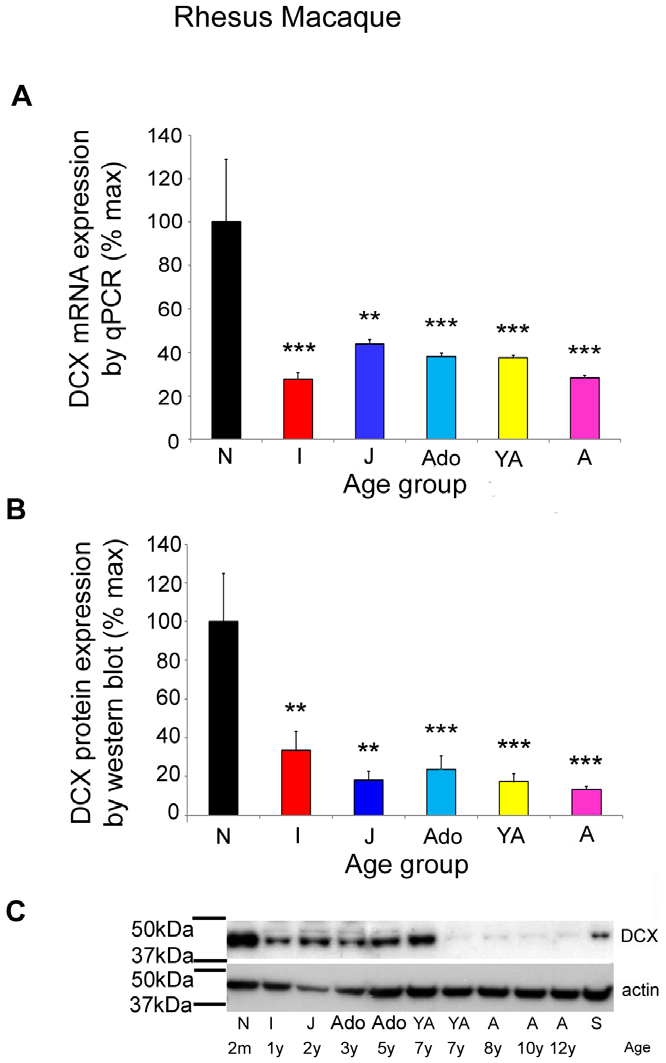
Doublecortin (DCX) is downregulated in the DLPFC of rhesus macaque brain during postnatal development. (**A**) mRNA expression profile of DCX (mean ± SEM) across chronological age by qPCR (DCX mRNA expression was normalised to the geometric mean of three housekeeper genes) and (**B**) DCX protein expression (mean ± SEM, normalised to β-actin). (**C**) Representative western blot for DCX and β-actin in cases from different developmental age groups. **p<0.005, ***p<0.001 compared to neonate. Black, neonates (N); red, infants (I); dark blue, juveniles (J); light blue, adolescents (Ado); yellow, young adults (YA); pink, adults (A). Pooled standard (S).

In rhesus macaque, qPCR analysis revealed a similar reduction in DCX expression over development to that found in humans, although it was not as marked. An approximate reduction of 77% in DCX mRNA expression was seen between neonate and adult rhesus macaques (ANOVA F(5, 39) = 5.74, p<0.0005, Figure 3A). Western blot analysis revealed approximately an 86% reduction in DCX protein expression between neonate and adult rhesus macaques (ANOVA F(5,39) = 5.6132, p<0.0005; Figure 3B) with a band at the predicted size of ∼40 kDa (Figure 3C). A significant difference was noted in both DCX mRNA and protein expression levels between neonates and the other developmental age groups (p<0.001 for mRNA and p<0.005 for protein) and a marked reduction in immunointensity on the Western blot can be seen in some individuals 8 years and older.

**Figure 4:**
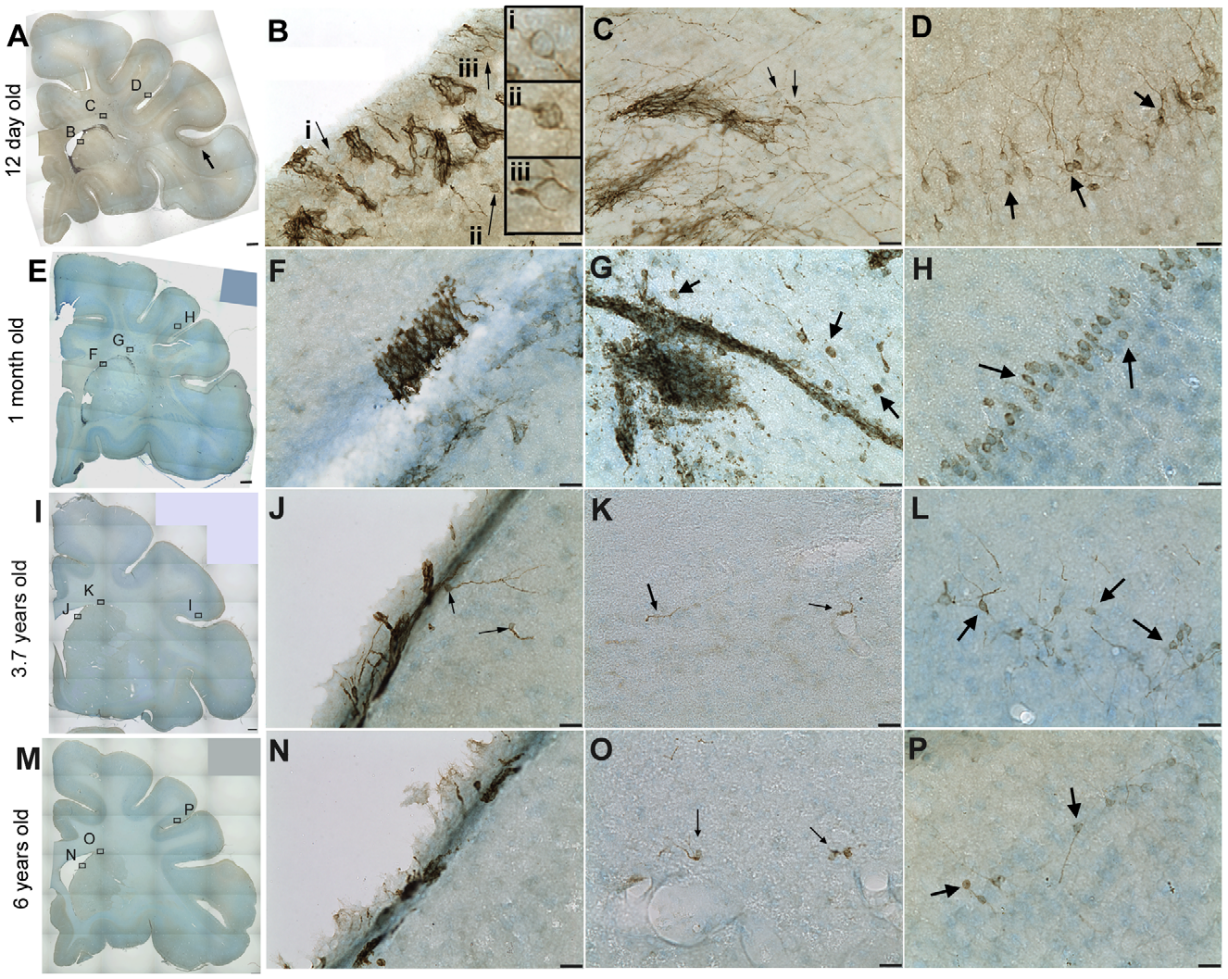
Doublecortin (DCX) is highly expressed in infant and expression continues into adulthood in rhesus macaque. In a 40 µm coronal section of a 12 day old rhesus macaque brain (**A**–**D**) DCX immunoreactivity is abundant around the lateral ventricle, particularly at the dorsal and ventral ends and in layer II of the cortex, particularly around the inferior arcuate sulcus (arrow) and orbital cortex. DCX cells are also present around the lateral ventricle in a 1 month old (**E**–**H**), 3.7 year old (**I**–**L**), and 6 year old (**M**–**P**) rhesus macaque brain. Higher power images show DCX positive cells and fibre plexus in the subventricular zone (**B**, **F**, **J**, **N**), DCX immunoreactivity in clusters in the white matter dorsal to the lateral ventricle in young brains (**C**, **G**), and DCX immunoreactivity in several cells or processes in the white matter adjacent to the dorsal ventricle in 3.7 and 6 year old brains (arrows **K**, **O**) and DCX immunoreactivity in layer II cells in the principal sulcus (with pial surface at top of the image in **D**, **L** and top-left of image in **H**, **P**). Scale bar = 1 mm (**A**, **E**, **I**, **M**), 20 µm (**B**–**D**, **F**–**H**, **J**–**L**, **N**–**P**).

**Figure 5:**
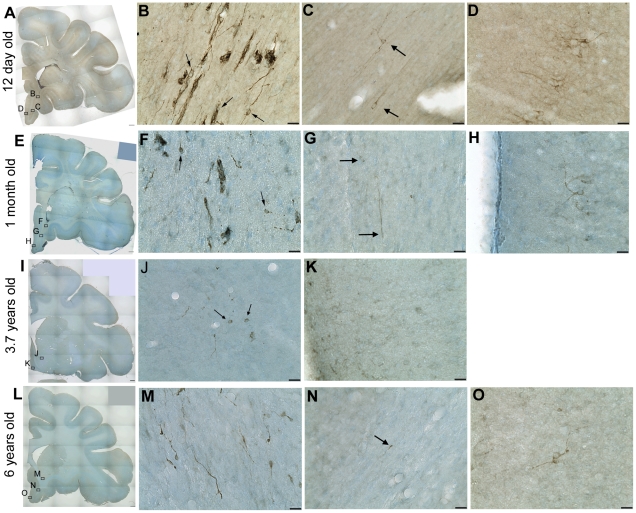
Doublecortin (DCX) expression in the developing rhesus macaque rectus gyrus. DCX immunoreactivity in a 40 µm coronal section from a 12 day old (**A**–**D**), 1 month old (**E**–**H**), 3.7 year old (**I**–**K**), and 6 year old (**L**–**O**) rhesus macaque brain. DCX immunoreactivity is abundant around the lateral ventricle, and in clusters and chains of cells ventral to the ventricle in the 12 day old (**B**) and 1 month old (**F**) animal. DCX cells (arrows) are also present ventral to the ventricle in a 3.7 year old (**J**) and a 6 year old animal (**M**). DCX positive cells are also present in the gyrus rectus of young animals (arrows **C**, **G**) and processes in the adult white matter (arrow **N**) and layer II cells in the gyrus rectus (**D**, **H**, **K**, **O**) (pial surface on left). Scale bar = 1 mm (**A**, **E**, **I**, **L**), 20 µm (**B**–**D**, **F**–**H**, **J**, **K**, **M**–**O**).

Although DCX immunoreactivity was not reliably detected in fresh frozen human tissue (including in a neonatal brain, using antibodies in Table S3, data not shown), DAB immunohistochemistry for DCX was successful on perfused, fixed rhesus macaque brain sections. Dense DCX positive cells and fibre plexus were observed across the different developmental ages. In the 12 day old rhesus macaque brain, DCX immunoreactivity was present in the white matter and particularly robust around the ventricle with clusters of DCX positive cells apparent in the VZ/SVZ on both the dorsal and ventral sides (Figure 4A). Although it is difficult to determine the morphology of individual cells in this area due to the density of DCX+ cells, many cells elaborate processes to the ventricle and/or into surrounding tissue (Figure 4B). In the neonatal rhesus macaque brain, large masses of DCX+ cells were also noted at the dorsal and ventral ends of the ventricle and clusters of cells with long processes were also present in the white matter around these masses (Figure 4C, Figure 5B) and many DCX+ cells (∼8 µm diameter, mostly with one or two long processes) were present in layer II of the cortex in the principal sulcus (Figure 4D), gyrus rectus (Figure 5D) and particularly around the inferior arcuate sulcus and lateral orbital sulcus. DCX positive cells were also observed in the corpus callosum (not shown). In the 1 month-old animal, intense DCX staining was also noted around the ventricle (Figure 4E), particularly in immunopositive patches on the dorsal and ventral sides (Figure 4F). Clusters and chains of DCX+ cells were present in the white matter dorsal and ventral to the ventricle (Figure 4G, Figure 5F) and a population of DCX+ cells was also present in layer II of the cortex at 1 month of age (Figure 4H, Figure 5H).

In the adolescent rhesus macaque (3.7 years), small DCX+ clusters of cells could be detected around the ventral ventricle (Figure 4J), however clusters of DCX+ cells dorsal or ventral to the ventricle were absent, but some individual immunopositive cells/processes could be seen in the white matter dorsal to the ventricle and caudate (Figure 4K). DCX+ cells were still present in layer II of the cortex, although less dense and with less elaborate processes than earlier in life (Figure 4L, Figure 5K). Similarly, in the adult rhesus macaque brain (6 years), there were relatively sparse DCX+ cell clusters around the ventral ventricle (Figure 4N) and few DCX+ cells in the white matter around or adjacent to the ventricle (Figure 4O, Figure 5M) and some DCX+ cells with 1–2 processes were detected in layer II of the cortex (Figure 4P, Figure 5O).

### IWMNs express markers of migration in the rhesus macaque brain

**Figure 6:**
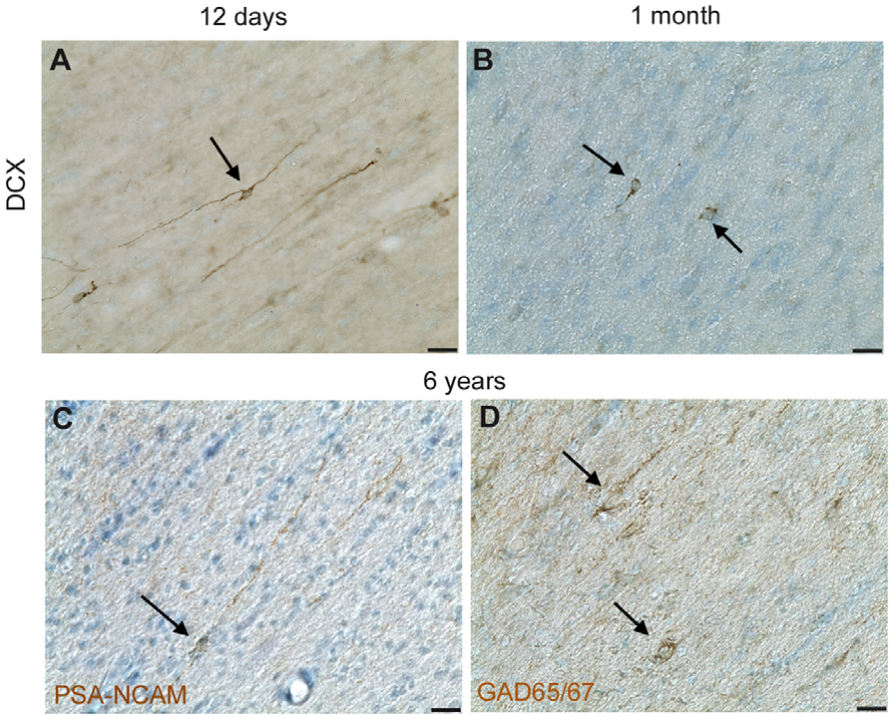
Non-human primate interstitial white matter neurons (IWMNs) express markers of migrating neurons and interneurons. Some individual IWMNs were immunopositive for DCX in 12 day old (arrows **A**) and 1 month old (arrows **B**) brains. PSA-NCAM immunoreactivty was apparent in some white matter neurons and their processes in the adult (40 µm sections) (arrows **C**). Some IWMNs were also positive for GAD65/67 immunoreactivity in the adult (arrows **D**) and there was also diffuse GAD65/67 immunoreactivity in the white matter of adults. Scale bars = 20 µm.

**Figure 7:**
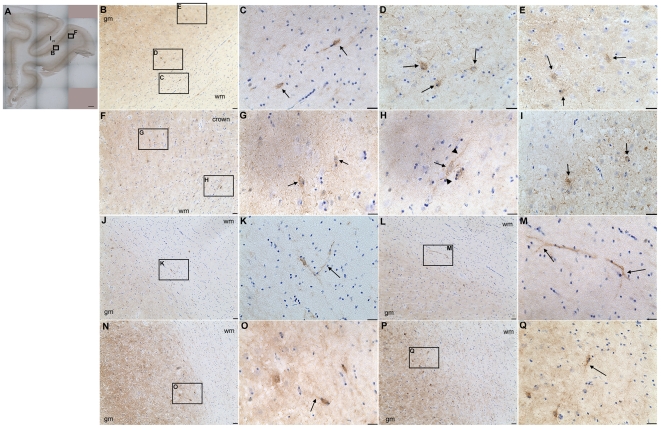
PSA-NCAM is expressed in multiple white matter neurons and immature cortical neurons in the adult rhesus macaque. Representative photos of DAB immunohistochemistry for PSA-NCAM in 14 µm coronal sections show diffuse immunoreactivity in layer IV of the cortex in the frontal pole of the adult (6.5, 7.6 and 9.6 years) rhesus macaque brain (**A**). Higher power images show multiple PSA-NCAM+ cells are also present in the white matter under the principal sulcus (**B**, **C**, **J**–**M**), and the numerous immunopositive cells are detected in the deeper cortical layers (**B**, **D**, **E**, **N**–**Q**) as well as layer II of the principal sulcus (**I**). Additionally, many PSA-NCAM+ cells could be seen in the white matter near the crown of the gyrus, often with elongated cell bodies (arrows in **G**) and some with one or two processes (arrowheads in **H**) along the long axis of the cell body (arrow in **H**). Some PSA-NCAM+ processes in the white matter were orientated on an angle to the pial surface (**J**, arrows in **K**), or may have bifurcated (**L**, arrows in **M**), and some PSA-NCAM+ cells with elongated cell bodies and a short process directed toward the pial surface were present in the deep cortical layers (arrows in **O**, **Q**). Scale bars: 1 mm (**A**), 40 µm (**B**, **F**, **J**, **L**, **N**, **P**), 20 µm (**C**–**E**, **G**–**I**, **K**, **M**, **O**, **Q**). gm = grey matter, wm = white matter.

Individual DCX+ cells with elongated cell bodies and processes orientated parallel to the pial surface were present in the white matter between sulci leading toward the principal sulcus (Figure 6A) as well as to the gyrus rectus (Figure 5C) in the 12 day old animal, and some DCX+ cells could be detected in the white matter between sulci, with somewhat less elaborate processes in the 1 month old rhesus macaque brain (Figure 6B). Distinct DCX+ cells were not observed in the white matter between sulci in the 3.7 year old or 6 year old brains; however, immunoreactivity for PSA-NCAM was observed on cells and processes in the white matter in 3.7 year old and 6 year old animals (Figure 6C), suggesting that these cells may be migrating neurons in the white matter of the adolescent and adult rhesus macaque. We confirmed this observation in an additional three adult animals (6.5, 7.6 and 9.6 years old), where we observed PSA-NCAM immunoreactivity in fresh-frozen tissue sections (Figure 7A). Many PSA-NCAM immunopositive cells were present in the white matter under the principal sulcus with elongated cell bodies and a leading and/or trailing process parallel to the pial surface (Figure 7B, C; enlarged in Figure S2). Many elongated PSA-NCAM positive cells were also directed toward the crown of the gyrus (Figure 7F–H), some with unipolar or bipolar processes oriented radially towards the crown, across the thickness of the grey matter at the crown (arrowheads in Figure 7H). Interestingly, we also observed some PSA-NCAM positive cells with a process that was directed on an angle to the pial surface, toward the grey matter (Figure 7J, K) or possibly bifurcated (Figure 7L, M). PSA-NCAM+ cells were also in the deeper layers of the cortex with a short process directed on an angle, or perpendicular to the pial surface (arrows in Figure 7N–Q). Immunoreactivity was diffuse in layer IV and several round, multipolar and elongated, unipolar/bipolar PSA-NCAM positive cells (∼8–15 µm diameter) were present in the deeper layers of the cortex (Figure 7D, E). In cortical layer II some smaller round (∼7–12 µm), PSA-NCAM cells whose morphology was difficult to define due to diffuse immunoreactivity that may be associated with processes were also present (Figure 7I).

### Rhesus macaque white matter neurons express GABAergic markers

**Figure 8:**
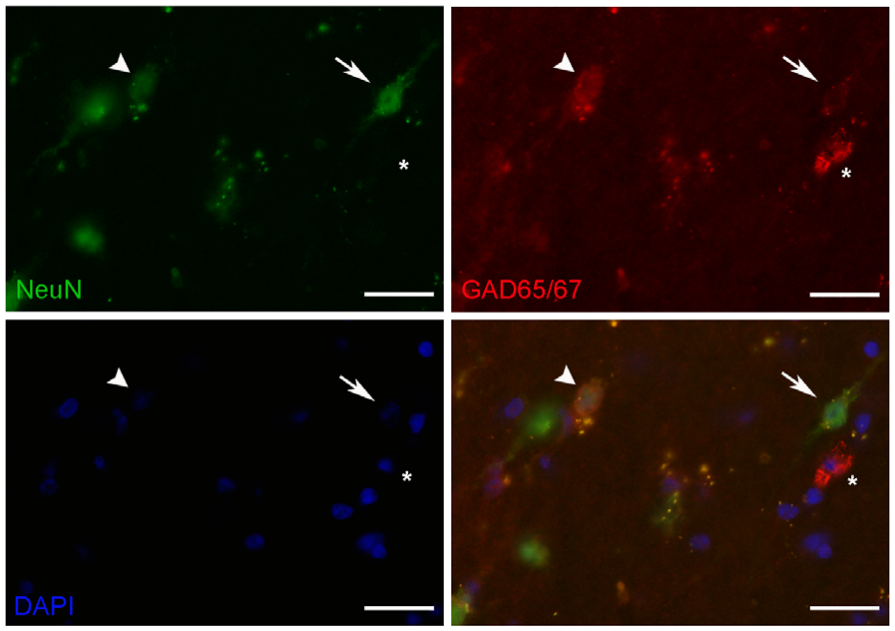
NeuN positive IWMNs express GABAergic markers. Double-label immunofluorescence in adolescent (4.5 year old) rhesus macaque frontal pole coronal 14 µm sections shows co-localisation of NeuN (green) with GAD65/67 (red) in white matter neurons (DAPI staining for nuclei in blue). Some neurons show faint immunoreactivity for GAD65/67 (arrow) and some neurons express relatively more GAD65/67 and less NeuN (arrowhead) or no NeuN (asterisk). Scale bars = 25 µm.

In the rhesus macaque brain, cells in the white matter between sulci were positive for GAD65/67, the rate limiting enzyme required for the synthesis of GABA, being present in the cell body and processes, suggesting that these white matter cells may be GABAergic neurons (Figure 6D), and we demonstrate co-localisation of GAD65/67 immunoreactivity in NeuN+ IWMNs in the principal sulcus of adolescent (4.5 years) rhesus macaques (n = 3) (Figure 8). GAD65/67 immunoreactivity was present in the majority of NeuN+ cells, however some GAD65/67+ cells displayed intense GAD65/67 immunoreactivity and less intense or no NeuN immunoreactivity (Figure 8, arrow head and asterisk).

### Expression of DCX mRNA in schizophrenia

**Figure 9:**
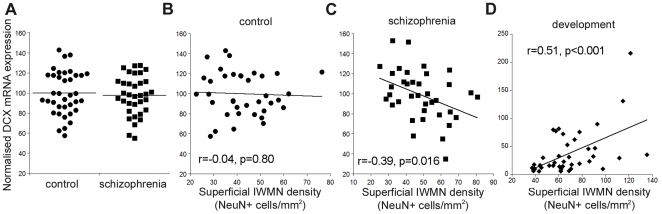
Doublecortin (DCX) is unaltered in schizophrenia cortex but related to white matter neuron (IWMN) density. DCX mRNA expression [normalised to the geometric mean of four housekeeping genes, shown as a % of control mean for control and schizophrenia groups (bar = mean)] was not altered in the brains of people with schizophrenia (n = 37) compared to control subjects (n = 37, p = 0.5, co-varying for age, pH, PMI, and RIN) (**A**). (**B**) DCX mRNA expression did not correlate with the density of superficial IWMNs in controls, and was negatively correlated with superficial IWMNs in patients with schizophrenia (**C**). (**D**) In development there was a positive correlation between DCX mRNA expression (normalised to the geometric mean of four housekeeping genes, shown as a % of the mean expression of the neonate group) and superficial IWMN density.

qPCR was used to determine expression of DCX mRNA in total RNA from the DLPFC of patients with schizophrenia/schizoaffective disorder (n = 37) and controls (n = 37). Analysis of covariance was then performed with DCX mRNA and diagnosis, co-varying for age, pH, PMI and RIN. DCX mRNA did not display differences in expression in adult schizophrenia patients compared with adult controls, with mean schizophrenia expression being 97.4% of controls (ANCOVA F (1, 65) = 0.31, p = 0.56; Figure 9A). No significant differences in expression by gender (t = 1.1, df = 69, p = 0.28) or hemisphere (t = 0.79, df = 69, p = 0.43) were noted. Performing Pearson's correlation to clinical variables (age of onset, duration of illness, daily chlorpromazine mean, last recorded chlorpromazine dose, and lifetime chlorpromazine exposure), we did not detect any significant correlation between expression of DCX with disease demographics or neuroleptic exposure (r = −0.22 to 0.07, all p>0.21).

### Relationship between DCX mRNA and IWMN density is altered in schizophrenia

We have previously reported that superficial IWMN density is elevated in people with schizophrenia in this cohort [18]. Therefore, to examine the relationship between DCX mRNA expression in the grey matter and superficial IWMN density, qPCR data for DCX in the grey matter was correlated with superficial IWMN density [18]. In adult controls from the schizophrenia cohort aged 18–78 years, there was no correlation of DCX mRNA with the density of superficial white matter neurons (r = −0.04, p = 0.80, Figure 9B); however , DCX mRNA expression was negatively correlated with superficial IWMN density in patients with schizophrenia (r = −0.39, p = 0.016, Figure 9C) [19]. In the human developmental cohort, in stark contrast, there was a positive relationship between grey matter DCX mRNA and superficial IWMN density (r = 0.51, p = 7.0×10^−4^, Figure 9D).

## Discussion

Our data suggest that the arrival of new neurons may play a significant role in the protracted postnatal development of the prefrontal cortex and, consequently, the behavioural and cognitive development associated with this region [52]. There is growing evidence to support the presence of immature neurons in the postnatal and adult primate neocortex; however, the origin of these cells is currently unclear. Our finding that DCX, a marker of immature neurons, is highly expressed in the first few years of life and continues to be expressed at lower levels in adulthood supports the hypothesis that the arrival of new, immature neurons into the DLPFC and gyrus rectus in postnatal life contributes to overall growth and maturation or attainment of abilities and/or learning. The observation of DCX+ cells surrounding the ventricle persisting into adulthood, the developmental down-regulation of IWMN density, and the presence of many PSA-NCAM+ cells in the white matter and cortex of adult non-human primate brains suggests that migration of immature neurons from the SVZ to the cortex, or to the white matter subjacent to the cortex could last for several years after birth in primate, and even into adulthood.

### New neurons could contribute to postnatal growth of the cortex

These findings demonstrate high expression of the neuronal migration marker DCX and high IWMN density soon after birth. DCX expression then dramatically declines in the first two years of life, and while cell death may account for some of the decrease in IWMN density, it is unlikely to explain the whole decrease, as very few nuclei are TUNEL positive early in human postnatal development [37]. Our observations replicate the expression of DCX in the human occipital cortex [20] and follow a similar developmental trajectory to that described for PSA-NCAM in the prefrontal cortex [53], supporting a developmental down-regulation of migrating neurons in postnatal life in the human cortex. This decrease in IWMN density is unlikely to be explained solely by a dilution effect caused by the expanding cortical volume and surface area during early development [41], [54]. The human brain increases in volume from birth to approximately 5 years of age [38], [39], [40], [41] and the brain weight nearly doubles from the neonate to the infant stage [55], [56]. Taking into account the expansion of the cortex and subcortical white matter over the first few years of life, the lack of significant change in IWMN density that we report between neonates and infants may even support an increase in the number of putatively migrating neurons at these early developmental time points. Indeed, cortical growth, particularly in the grey matter which increases across childhood [57], [58], may, in part, represent the arrival of new neurons in early postnatal development, as an increase in neuronal density has been reported in the human cerebral cortex between 15 months and 6 years of age [54].

### New cortical neurons may originate from the adult SVZ

In the early human brain, the majority of interneuron genesis takes place in the ganglionic eminence, however at 20 gestational weeks (gw) many calretinin positive interneurons are also present in the cortical VZ/SVZ [59] and by 25 gw the majority of interneurons in the cortical plate (65%) are Mash1 positive [60], suggesting they may have originated in the dorsal VZ/SVZ [59], [60], [61], [62]. We have previously shown neuronal clusters positive for ErbB4, PSA-NCAM and TuJ1 that appear to be migrating away from the ventral SVZ in the developing, postnatal human brain which may be the source of origin of new neurons in the cortex [36], [37]. Our finding here of numerous DCX positive cell clusters in the ventral SVZ of the monkey, even in the adult, is indicative of an immature population of cells derived from the ventral VZ/SVZ and is consistent with this notion.

It has been suggested that cells that arise from the ventral SVZ may take a route like that of the rostral migratory stream to locations such as the amygdala and prefrontal, parietal, piriform, entorhinal and temporal cortices in rodents, rabbits, and non-human primates [63], [64], [65], [66], [67], [68], [69]. This observation has recently been extended to the adult human, where Wang and colleagues showed continued proliferation of neuroblasts in the anterior ventral SVZ and immature cells expressing DCX and PSA-NCAM in the RMS [33]. Further to this, Inta and colleagues used time-lapse imaging of transgenic 5-HT_3_-EGFP mice to demonstrate the postnatal migration (in juveniles) of neurons from the SVZ to the cortex and subcortical regions, demonstrating DCX expression in these cells and an ultimate GABAergic phenotype [70]. The abundance of DCX positive cells around the ventricle, and the presence of DCX and PSA-NCAM positive neurons in the white matter of the principal sulcus in infant rhesus macaques that we report here provide further support of postnatal migration of neurons from the SVZ to the cortex.

Although we did not find any DCX immunopositive cells in the white matter of adult primates, the expression of PSA-NCAM by rhesus macaque adult IWMNs and their bipolar, tangential morphology suggests that these immature cells may be migrating through the white matter. It is possible that DCX expression is below the level of detection in these cells, or that there may be another DCX-like molecule being expressed due to possible molecular redundancy as has been suggested with DCL and DCLK [25], [26]. We suggest that neurons migrating from the SVZ, or generated in the white matter may contribute to immature PSA-NCAM expressing neurons in the deeper layers of the cortex as we observed several PSA-NCAM+ cells that appeared to be “turning” from the white matter with a process directed toward the grey matter and many unipolar and bipolar PSA-NCAM positive cells in the deeper cortical layers oriented toward the crown of the gyrus in the adult rhesus macaque cortex.

### Immature neurons in the adult cortex

Our observations indicate that expression of DCX mRNA is persistent at detectable levels in the grey matter throughout adult life. In the adult, neurons that are positive for DCX protein and/or PSA-NCAM have been reported in the cortex, particularly layer II, of several species including rodents, cats, non-human primates, and humans [31], [63], [67], [71], [72], [73], [74], [75], [76]. It is thought that these cells are immature neurons due to their co-localisation with neuronal markers such as TUC-4 (a neuronal lineage marker) [72], TuJ1 [76] and expression of NeuN in some cells [31], [72], [76], [77]. Interestingly, the density of these immature neurons is reduced in ageing [31], [78], which may indicate that these cells become depleted over time [79]. There is, however, controversy as to whether these DCX+ cells may become GABAergic or glutamatergic as some studies report expression of GABA, GAD67, calretinin and parvalbumin with DCX [31], [76], while others report no co-localisation with GABAergic markers, and the expression of Tbr-1 (a transcription factor of dorsal SVZ neurons) in these immature DCX+ neurons [72], [74], [77]. Varea and colleagues show immature DCX+/PSA-NCAM+ neurons in layer II express Tbr-1, although BrdU labelling in adult cats indicates that the majority of PSA-NCAM+ cells may not be born in adult life [77], and, in the rodent, the birth of layer II cells peaks around E15.5 [72]. However, new neurons in the adult are expected to be less than 0.03% of total neurons (as reviewed by [80]), and thus may be difficult to detect with the BrdU regimens used (2–4 injections of BrdU over a two day period). A further population of larger PSA-NCAM positive cells are also found in deeper cortical layers (as we find here), some of which expressed GAD67 and/or calbindin and calretinin, suggesting that these deeper cortical immature neurons may be GABAergic [77]. This would be consistent with the inhibitory nature of the IWMNs we report here in the rhesus macaque brain (GAD65/67+) and our previous observation of somatostatin and neuropeptide Y expression in human IWMNs [18].

The high expression of grey matter DCX in the first few years of life is consistent with DCX involvement in cortical growth and it follows that differentiation of newly arrived cells may result in delayed up-regulation of the interneuron markers that are late developing. Indeed, we have recently reported within the same cohort that the most dramatic changes, either up-regulation or reduction, in mRNA expression of multiple biochemical markers of interneurons occur within the first five postnatal years in the human DLPFC [44]. Protracted increases in interneuron markers parvalbumin, cholecystokinin, calbindin and vasoactive intestinal peptide in the DLPFC over the first years of life were shown [46], that are reciprocal to the reduction in DCX, implying that some immature migrating neurons may down-regulate DCX and up-regulate markers of differentiated interneurons in the human frontal cortex. This is consistent with other reports, such as up-regulation of parvalbumin in cartridges and neurons, and calbindin positive neurons that display protracted development in the primate [81], [82], and in human frontal [83] and entorhinal cortex [84].

### DCX in schizophrenia

Putative candidate schizophrenia genes like neuregulin/ErbB4 and reelin have important roles in migration of new neurons, and some studies have implied a reduction in neurogenesis in the schizophrenic brain [44], [45]. While we have previously reported an increase in the density of IWMNs in schizophrenia using this cohort [18], we do not find a change in DCX mRNA in the DLPFC in schizophrenia here. However, even though DCX is expressed in the cortex of the adult it may be an imperfect marker for neuronal migration in the adult due to its relatively low expression level and variable intensity in layer II neurons. This may be supported by the wide spread in DCX mRNA expression in the normal adults, and variable immunointensity within a given neuron, such that DCX mRNA could be regulated by factors like cell activity which may differ between individuals. Additionally, the age of individuals in the control and schizophrenia population (spanning from 18–80 years) may make subtle alterations in DCX mRNA expression difficult to detect.

Interestingly, while in the normal adult brain there is no correlation between DCX grey matter mRNA and IWMN density, in people with schizophrenia there is a negative correlation, such that individuals with less DCX mRNA in the grey matter tend to have more subjacent IWMNs. This change parallels our previous finding of reduced interneuron marker mRNA (somatostatin) being correlated with increased IWMN density in schizophrenia [18], and is consistent with a failure of migration of some newly born neurons to the cortex in development. We hypothesise that NeuN expression may overlap with that of some immature markers, such as DCX (as this also occurs in layer II) [77], [78], as immature neurons down-regulate immature neuron markers and up-regulate NeuN while they begin to differentiate [18], [85]. Therefore, NeuN expressing neurons in the white matter would correlate with the increase in DCX in the grey matter at a time of high neuronal migration. In the adult controls, there appears to be an uncoupling of DCX mRNA expression and IWMN density that could be due to low levels of neuronal migration and the variable presence of DCX in many layer II neurons. If developmental neuronal migration is deficient in the brains of people with schizophrenia, this could lead to an accumulation of IWMNs under the cortex, and higher numbers of IWMNs in individuals with schizophrenia that may constrain the immature neurons reaching the cortex and drive the negative correlation between IWMN density and DCX mRNA expression in the disease state.

Although adult cortical neurogenesis is controversial, our results support the hypothesis that neuronal migration to the cortex may be robust in the early postnatal primate brain. We also demonstrate that molecules associated with immature neurons, neuronal migration and/or plasticity can still be found in the adult primate brain in grey matter and in the white matter, and we suggest that migration of immature inhibitory neurons continues to occur into the adult primate frontal cortex albeit at much lower levels. Our results support seminal findings of Gould and others [63], [64], [65] that raise the question as to whether or not the olfactory bulb is the sole destination of newly born neurons of the primate SVZ. While we did not detect a change in the expression of DCX in the brains of people with schizophrenia, more sensitive or direct methods may be required to detect altered migration of neurons in the disease (which would likely affect a small population of the total number of neurons in the cortex) and further lines of evidence, such as altered IWMN density and positioning, and the involvement of several schizophrenia susceptibility genes in neuronal migration indicate that altered neuronal migration may be implicated in schizophrenia pathology. Understanding postnatal neurogenesis and persistent migration of immature neurons in the juvenile and adult brain suggests that cortical neurogenesis may represent an important therapeutic target for intervention in schizophrenia.

## Supporting Information

Figure S1 **Interstitial white matter neuron (IWMN) density.** Representative photomicrograph showing grey matter and white matter boundary (line) used for quantification of superficial IWMNs. The representative image shows DAB immunohistochemistry for NeuN in a normal adult human brain. Some examples of IWMNs are indicated with arrows.
(TIF)

Figure S2 **Non-human primate interstitial white matter neurons (IWMNs) express PSA-NCAM.** PSA-NCAM immunoreactivity was apparent in multiple white matter neurons in adult rhesus macaques at several ages. PSA-NCAM + INWMs in (**A**) 6.5 year old, (**B**) 7.6 year old and (**C**) 9.6 year old animals. Some examples of PSA-NCAM+ IWMNs are indicated with arrows. Scale bars = 50 µm.
(TIF)

Table S1 **Summary of human developmental cohort used for experiments.** PMI = post-mortem interval, RIN = RNA integrity number, M = male, F = female, IWMN = interstitial white matter neuron.
(XLSX)

Table S2 **Antibodies used in DAB immunohistochemistry.** DAB = 3,3′–diaminobenzidine, GAD = glutamic acid decarboxylase, PSA-NCAM = polysialyated neuronal cell adhesion molecule.
(XLSX)

Table S3 **Antibodies used for DCX in fresh-frozen tissue pilot.**
(XLSX)
