# Supplementary figures and images for: Developmental Patterns of Doublecortin Expression and White Matter Neuron Density in the Postnatal Primate Prefrontal Cortex and Schizophrenia
Source: PLoS One. 2011 Sep 26;6(9):e25194. doi: 10.1371/journal.pone.0025194 (PMC3180379; doi:10.1371/journal.pone.0025194)

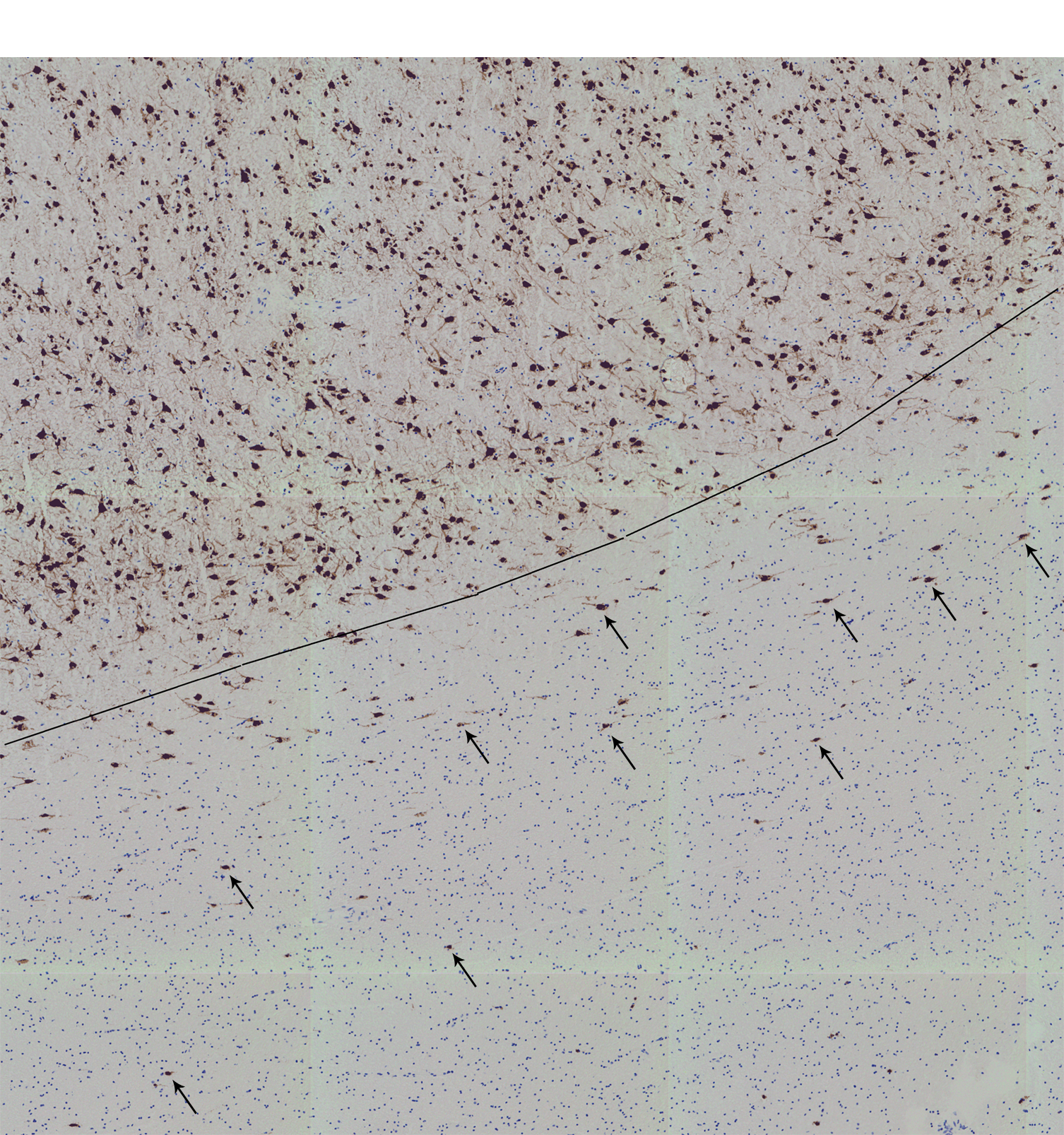

Supplement: Figure S1 — Interstitial white matter neuron (IWMN) density. Representative photomicrograph showing grey matter and white matter boundary (line) used for quantification of superficial IWMNs. The representative image shows DAB immunohistochemistry for NeuN in a normal adult human brain. Some examples of IWMNs are indicated with arrows. (TIF) [file pone.0025194.s001.tif]

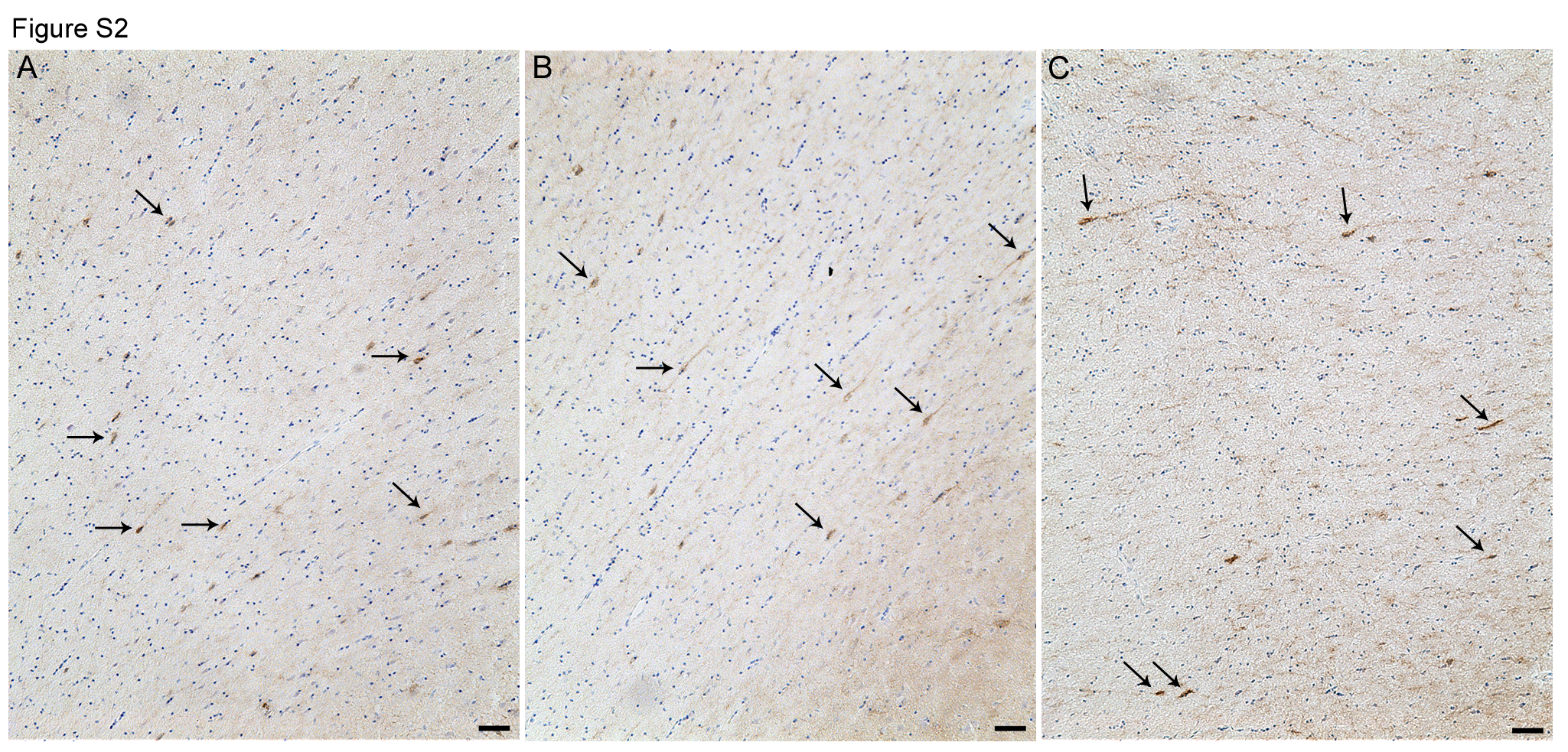

Supplement: Figure S2 — Non-human primate interstitial white matter neurons (IWMNs) express PSA-NCAM. PSA-NCAM immunoreactivity was apparent in multiple white matter neurons in adult rhesus macaques at several ages. PSA-NCAM + INWMs in (A) 6.5 year old, (B) 7.6 year old and (C) 9.6 year old animals. Some examples of PSA-NCAM+ IWMNs are indicated with arrows. Scale bars = 50 µm. (TIF) [file pone.0025194.s002.tif]
